# Tumor homologous recombination deficiency assays: another step closer to clinical application?

**DOI:** 10.1186/s13058-014-0409-7

**Published:** 2014-07-14

**Authors:** Shane R Stecklein, Priyanka Sharma

**Affiliations:** Department of Pathology and Laboratory Medicine, University of Kansas Medical Center, 3901 Rainbow Boulevard, MS1016, Kansas City, KS 66160 USA; Division of Hematology/Oncology, Department of Internal Medicine, University of Kansas Medical Center, 2330 Shawnee Mission Parkway, MS5003, Westwood, KS 66205 USA

## Abstract

Inherited and acquired defects in homologous recombination, a phenotype termed ‘*BRCA*ness’, may lend to therapeutic exploitation in breast cancer. To this end, development and clinical evaluation of platforms to identify signatures of *BRCA*ness are of immense interest. In this issue of *Breast Cancer Research*, Vollebergh and colleagues report that a *BRCA*-like array comparative genomic hybridization (aCGH) genomic instability signature is associated with benefit from high-dose cyclophosphamide-thiotepa-carboplatin chemotherapy. We discuss the strengths and weaknesses of this study and consider the clinical significance and applicability of this aCGH *BRCA*ness signature in the context of other existing homologous recombination deficiency detection platforms.

In this issue of *Breast Cancer Research*, Vollebergh and colleagues [1] report on the predictive capacity of an array comparative genomic hybridization (aCGH) signature resembling *BRCA1*- and *BRCA2*-mutant breast cancers. The *BRCA1* and *BRCA2* genes are required for DNA double-strand break (DSB) and interstrand crosslink repair by homologous recombination (HR) [2,3]. Pre-clinical and clinical data suggest that cancers arising in *BRCA1* and *BRCA2* mutation carriers are particularly responsive to agents that lead to DSBs (for example, platinum salts and poly(ADP-ribose) polymerase (PARP) inhibitors) [4-6]. Although inherited mutations in *BRCA1* and *BRCA2* account for only 5 % to 10 % of all breast cancers, these and other genes which operate in the HR pathway may be altered by mutation, rearrangement, DNA methylation, or attenuated mRNA expression, resulting in impairment of HR in a significant proportion of patients with breast cancer. Inactivation of HR by these mechanisms in *BRCA*-wild-type cancers (a phenotype termed ‘*BRCA*ness’) may induce a similar hypersensitivity to DNA-damaging agents which can be therapeutically exploited. To this end, development and clinical evaluation of platforms to identify signatures of *BRCA*ness have recently been a subject of intense investigation, especially in triple-negative breast cancer (TNBC), a subtype thought to be enriched for *BRCA*ness [7-9].

Vollebergh and colleagues retrospectively assessed a subset of HER-2-negative tumor specimens from a randomized clinical trial that compared five cycles of 5-fluorouracil-epirubicin-cyclophosphamide (FE_90_C) with four cycles of FE_90_C followed by high-dose cyclophosphamide-thiotepa-carboplatin (HD-CTC) with autologous stem cell support in patients with at least four positive axillary lymph nodes. The *BRCA1*- and *BRCA2*-like aCGH profiles were based on previous work by this group and were generated on 249 HER-2-negative formalin-fixed paraffin-embedded (FFPE) tumor samples [10]. Overall, the *BRCA*-like^cgh^ profile was detected in 32 % (12 % *BRCA1*-like, 15 % *BRCA2*-like, and 5 % both *BRCA1*- and *BRCA2*-like) of the study cohort and predicted benefit from HD-CTC in both TNBC and hormone receptor-positive breast cancer. HD-CTC was associated with improved overall survival compared with FE_90_C in patients with *BRCA*-like^cgh^ tumors but not in patients with non-*BRCA*-like^cgh^ tumors. As expected, a significant proportion (76 %) of TNBCs harbored the *BRCA*-like^cgh^ profile. Interestingly, 28 % of hormone receptor-positive tumors also demonstrated the *BRCA*-like^cgh^ profile. This aCGH platform is attractive as it allows detection of genomic instability resulting from HR deficiency without requiring identification of the precise molecular etiology. If validated in other cohorts, this assay could potentially identify patients destined to benefit from intensified DNA-damaging therapy.

Despite the future potential of this aCGH platform as a predictive marker, the present study has important limitations. First, the HD-CTC regimen included both carboplatin and intensified alkylating chemotherapy (cyclophosphamide and thiotepa) and therefore it remains unclear whether the benefit observed in the *BRCA*-like^cgh^ patients within the experimental cohort was due to the platinum or the intensified alkylating agents or both. This is a significant confounder and complicates interpretation of these data. Additionally, although this study included a high-risk population (at least four positive nodes), neither the experimental (HD-CTC) nor the control (taxane-devoid chemotherapy) regimens are considered standard for node-positive breast cancer in this era. Evaluation of this assay in cohorts treated with contemporary neoadjuvant chemotherapy regimens is encouraged.

Currently, a standard platform for detecting *BRCA*ness has not reached routine clinical application, but several functional assays besides the aCGH platform described by Vollebergh and colleagues have emerged. The homologous recombination deficiency (HRD) assay developed by Myriad Genetics, Inc. (Salt Lake City, UT, USA) evaluates tumor genome loss of heterozygosity, telomeric allelic imbalance (TAI), and large-scale state transitions, which are all indirect measures of tumor genomic instability. High HRD scores are highly correlated with defects in *BRCA1/2* and are associated with sensitivity to neoadjuvant platinum-based chemotherapy in TNBC [9,11]. Another study examining TAI in sporadic TNBC has shown that tumors with high levels of TAI respond better to neoadjuvant platinum chemotherapy [7]. In addition to exhibiting genomic instability, tumors with *BRCA*ness may exhibit characteristic gene expression patterns. Mulligan and colleagues [8] recently reported on a 44-gene DNA damage response deficiency signature (DDRDS) which was developed in cohorts enriched for germline *BRCA1/2* and Fanconi anemia mutations. The DDRDS is enriched for immune response-related genes and predicted favorable response to FE_90_C chemotherapy in both TNBC and hormone receptor-positive breast cancer. The aCGH, HRD, TAI, and DDRDS platforms are all compatible with FFPE tissues, making them suitable for prospective studies. Furthermore, all of these HR deficiency platforms suggest that the *BRCA*ness phenotype exists in at least half of patients with TNBC and one quarter of patients with hormone receptor-positive breast cancer, implying future predictive applicability to a large fraction of patients with breast cancer.

There is now renewed interest in the efficacy of platinum agents for the treatment of TNBC as recent randomized trials have demonstrated improvement in pathological complete response rates with the addition of neoadjuvant carboplatin to anthracycline/taxane-based chemotherapy [12-14]. HR deficiency assay assessments on the completed randomized neoadjuvant platinum trials are eagerly awaited and will help identify patients most likely to benefit from such an approach. Whether HR deficiency assays will predict benefit from PARP inhibitors in addition to DNA-damaging chemotherapy also remains to be seen.

Employing functional measures of HR pathway deficiency rather than relying on documented changes in specific genes should capture more patients who might benefit from DNA-damaging therapies, and Vollebergh and colleagues should be congratulated on developing one such assay. However, before HR deficiency assays can be used for medical utility, the recognized steps for incorporation of predictive biomarkers into clinical care (that is, additional validation studies and assessment within prospective randomized trials) have to be fulfilled [15]. If appropriately validated, HR deficiency assays could have tremendous impact on patient care by identifying patients most likely to benefit from DNA-damaging agents like platinum salts or PARP inhibitors or both.

In conclusion, considerable strides have been made in analytical development and early clinical validation of functional HR deficiency assays and these efforts bring us one step closer to the eventual utilization of *BRCA*ness as a guide to personalize treatment with DNA-damaging agents in patients with breast cancer.

## Abbreviations

aCGH: Array comparative genomic hybridization
DDRDS: DNA damage response deficiency signature
DSB: Double-strand break
FE_90_C: 5-fluorouracil-epirubicin-cyclophosphamide
FFPE: Formalin-fixed paraffin embedded
HD-CTC: High-dose cyclophosphamide-thiotepa-carboplatin
HR: Homologous recombination
HRD: Homologous recombination deficiency
PARP: Poly(ADP-ribose) polymerase
TAI: Telomeric allelic imbalance
TNBC: Triple-negative breast cancer
